# Trigeminal neuralgia: a practical guide

**DOI:** 10.1136/practneurol-2020-002782

**Published:** 2021-06-09

**Authors:** Giorgio Lambru, Joanna Zakrzewska, Manjit Matharu

**Affiliations:** 1 The Headache Service, Pain Management and Neuromodulation Centre, Guy's and St Thomas' Hospitals NHS Trust, London, UK; 2 Facial Pain Clinic, Eastman Dental Hospital, London, UK; 3 Pain Management Centre, University College London Hospitals NHS Foundation Trust, London, UK; 4 Headache and Facial Pain Group, UCL Queen Square Institute of Neurology, London, UK; 5 Headache and Facial Pain Group, National Hospital for Neurology and Neurosurgery, London, UK

**Keywords:** pain, trigeminal nerve, trigeminal neuralgia, headache

## Abstract

Trigeminal neuralgia (TN) is a highly disabling disorder characterised by very severe, brief and electric shock like recurrent episodes of facial pain. New diagnostic criteria, which subclassify TN on the basis of presence of trigeminal neurovascular conflict or an underlying neurological disorder, should be used as they allow better characterisation of patients and help in decision-making regarding medical and surgical treatments. MR imaging, including high-resolution trigeminal sequences, should be performed as part of the diagnostic work-up. Carbamazepine and oxcarbazepine are drugs of first choice. Lamotrigine, gabapentin, pregabalin, botulinum toxin type A and baclofen can be used either alone or as add-on therapy. Surgery should be considered if the pain is poorly controlled or the medical treatments are poorly tolerated. Trigeminal microvascular decompression is the first-line surgery in patients with trigeminal neurovascular conflict while neuroablative surgical treatments can be offered if MR imaging does not show any neurovascular contact or where patients are considered too frail for microvascular decompression or do not wish to take the risk.

## Introduction

Trigeminal neuralgia (TN) is characterised by recurrent, unilateral, brief (<1 s–2 min), very painful, electric shock-like pain episodes in the trigeminal distribution that are abrupt in onset and termination.

It is a highly debilitating disorder that impacts on basic human functions such as talking, eating, drinking and touching the face, thereby resulting in a poor quality of life. Epidemiological studies show increased anxiety and depression, with increased risk of suicide.^1^ This highlights the importance of prompt diagnosis, investigations and treatment.

## Epidemiology

The lifetime prevalence of TN is estimated to be 0.16%–0.3%,^2 3^ while the annual incidence is 4–29 per 100 000 person-years.^4–6^ It is more prevalent in women than in men (F:M ratio 3:2).^5 7^ The incidence increases with age, with a mean age of onset of 53–57 years and range of 24–93 years in adult series.^1 7^ Furthermore, a recent paediatric headache clinic of 1040 identified five children in the age range 9.5–16.5 years with TN.^8^

## Diagnostic criteria and classification

The International Classification of Headache Disorders third edition (ICHD-3) criteria for TN require recurrent paroxysms of unilateral facial pain restricted to the trigeminal distribution, lasting from a fraction of a second to 2 min, severe in intensity with an electric shock-like shooting, stabbing or sharp quality, and precipitated by innocuous stimuli (see box 1).^9^

Box 1 **International Classification of Headache Disorders edition 3 (ICHD-3) diagnostic criteria for trigeminal neuralgia^9^**

- Recurrent paroxysms of unilateral facial pain in the distribution(s) of one or more divisions of the trigeminal nerve, with no radiation beyond, and fulfilling criteria B and C.
- Pain has all of the following characteristics:
  1. Lasting from a fraction of a second to 2 min.
  2. Severe intensity.
  3. Electric shock-like shooting, stabbing or sharp in quality.
- Precipitated by innocuous stimuli within the affected trigeminal distribution.
- Not better accounted for by another ICHD-3 diagnosis.

**Figure 1:**
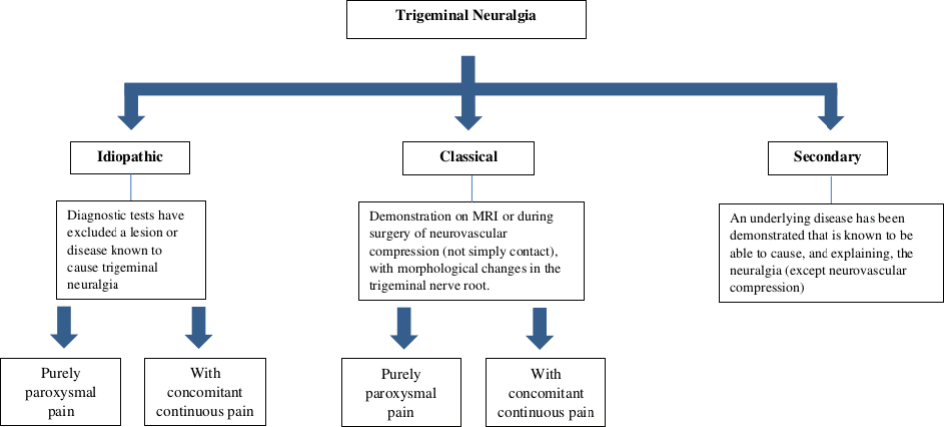
International Classification of Headache Disorders Edition 3 subclassification of trigeminal neuralgia.^9^

TN is further subclassified into classical, secondary or idiopathic, depending on the underlying cause (figure 1). The classical type, which is the most common and accounts for 75% of cases, is diagnosed when there is trigeminal neurovascular compression with morphological changes ipsilateral to the side of the pain, demonstrated either on MR imaging with appropriate trigeminal sequences or during surgery. Simple trigeminal contact without morphological changes is not sufficient to underpin such a diagnosis as this is a common neuroimaging finding in healthy people. Indeed, prospective trigeminal MR imaging studies have shown that on the symptomatic side, classical TN is associated with neurovascular compression with morphological changes (distortion, indentation, atrophy) while these morphological changes are rare on the asymptomatic side.^10^ The secondary type, accounting for approximately 15% of cases, is attributable to an identifiable underlying neurological disease (except trigeminal neurovascular compression) that is known to cause TN, such as cerebellopontine angle tumour, arteriovenous malformation and multiple sclerosis. Approximately 2% of people with multiple sclerosis have symptoms similar to those of TN.^11^ The idiopathic type, accounting for approximately 10% of cases, is diagnosed when no apparent cause for TN can be found.

Idiopathic and classical TN are further subclassified in groups with purely paroxysmal pain or with concomitant continuous pain (depending on the presence or absence of continuous or near continuous interictal pain).

## Clinical features

The ICHD-3 diagnostic criteria outline the cardinal features of the TN phenotype. However, the clinician’s appreciation of the detailed phenotype aids the diagnostic process particularly with regard to alerting to atypical features that warrant consideration of other diagnosis or further investigations.

### Laterality and site of pain

The right side of the face (60%) is affected more than the left side.^12^ Bilateral simultaneous pain in TN is rare (1.7%–5%) and more often these patients experience side-alternating unilateral pain paroxysms. In view of its rarity, bilateral simultaneous or side-alternating trigeminal paroxysmal pains should raise concern about an underlying neurological disorder or a non-neurological disorder affecting the cranium. It therefore warrants careful exclusion of secondary pathology.^13^ If investigations are normal, then idiopathic cases of constant or long-lasting bilateral trigeminal pain include: temporomandibular joints dysfunction, persistent idiopathic facial pain and rarely migraine with facial pain. In cases with paroxysmal short-lasting pain episodes, trigeminal autonomic cephalalgias such as short-lasting unilateral neuralgiform headache attacks (SUNHA) should be considered if pain is associated with cranial autonomic symptoms or idiopathic stabbing headache if the pain is predominantly in the ophthalmic (V1) trigeminal distribution.

The pain of TN most frequently affects the distribution of the maxillary (V2) and mandibular (V3) divisions of the trigeminal nerve, though approximately a quarter of the cases have ophthalmic (V1) division involvement.^7^

### Frequency and duration of attacks

The frequency and duration of TN attacks are highly variable. While the pain usually lasts from less than a second up to 2 min in the majority (74%), a significant minority reports attacks lasting 2–10 min.^14^ Furthermore, up to 70% of patients occasionally have series of paroxysms lasting up to 1 hour, which can cause diagnostic confusion.^7^ In patients with long-lasting attacks (>2 min) but with a phenotype otherwise consistent with TN, it is imperative to rule out other neuralgiform disorders. The number of attacks is highly variable even in the same patients and ranges from a few attacks to several hundred attacks daily; approximately 40% of patients report more than 10 attacks daily.^7^ Obtaining a good descriptive history of frequency and duration of attacks in short-lasting trigeminal neuralgiform pain conditions is often challenging. Using pain diagrams may help to clarify our definition of a single paroxysm as opposed to a group of paroxysms.^15^

TN follows a relapsing–remitting pattern in approximately two-thirds of patients but has a chronic pattern in the remaining one-third. Both the frequency and duration of the remission periods vary greatly, with the remission periods lasting months (37%) or years (63%).^7^

### Triggers and trigger zones

One of the hallmark clinical features of TN is the triggerability of the attacks by innocuous mechanical stimulation of the face and intraoral mucosa ipsilateral to the side of the pain. Around 91%–99% of patients report triggered attacks and these are often considered to be pathognomonic of TN.^7 16 17^ Patients usually report a mixture of triggered and spontaneous attacks, with 68%–98% of cases having spontaneous attacks. A complete lack of triggerable attacks should prompt careful assessment to exclude an alternative diagnosis including a trigeminal autonomic cephalalgia or craniofacial pathology.

Light tactile stimulation is the most potent trigger and, conversely, painful and thermal stimulation seems ineffective at eliciting pain in TN.^18^ Common triggers include light touch, talking, chewing, brushing teeth, washing or drying, drinking and shaving.^19^ Most patients have several trigger factors.^7 16^ The location of the pain does not always concord with the site of trigger zone.^19^ The most common trigger zones include the nasolabial fold, upper lip, lateral part of the lower lip, chin, cheek and the alveolar gingiva.^16^

### Refractory period

In most people with TN, a triggered attack is normally followed by a period of seconds or minutes during which further attacks cannot be provoked, a phenomenon called refractory period.^18^ This contrasts with the trigeminal autonomic cephalalgia, SUNHA, in which there is mostly no refractory period after exposure to a trigger.^20^

### Associated cranial autonomic symptoms

There are several case series of TN that describe cranial autonomic symptoms. This potentially poses a challenge differentiating TN from trigeminal autonomic cephalalgias, which are characterised by prominent cranial autonomic symptoms. Rasmussen described 98 out of the 229 (43%) patients in whom the pain was accompanied by facial autonomic symptoms including lacrimation (31%), rhinorrhoea (9%), hypersalivation (7%) and facial swelling/flushing (5%).^21^ A recent Danish study reported that 48 of 158 patients (31%) experienced ipsilateral cranial autonomic symptoms during attacks.^13^ In both these series, these symptoms were more likely to be reported by patients with ophthalmic division trigeminal pain (V1). In contrast, Sjaastad *et al* carefully dissected the phenotype of 19 patients with V1 TN and reported the occurrence of lacrimation (42%), conjunctival injection (16%) and rhinorrhoea (11%), but the cranial autonomic symptoms were mild in all patients.^22^

The series reporting TN with prominent cranial autonomic symptoms are in fact misdiagnosed cases of SUNHA; SUNHA is further subclassified as SUNCT (short-lasting unilateral neuralgiform headache attacks with conjunctival injection and tearing) in patients with both conjunctival injection and lacrimation, or SUNA (short-lasting unilateral neuralgiform headache attacks with cranial autonomic symptoms) in patients with at least one cranial autonomic symptom but not both conjunctival injection and tearing. From a practical perspective, if a patient with a TN phenotype has only mild and sporadic cranial autonomic symptoms, then the diagnosis of TN can be maintained, whereas if these autonomic symptoms are intense (eg, profuse lacrimation and rhinorrhoea), numerous (>1 of these symptoms) and consistently accompanying most attacks, then the patient should be diagnosed with SUNCT or SUNA. Other clinical features that can make a diagnosis of SUNHA more likely than TN in clinical practice include predominant pain in V1 trigeminal distribution, spontaneous-only attacks, absence of refractory period in triggered attacks and longer lasting attacks.

### TN that is purely paroxysmal or with concomitant continuous pain

TN with concomitant continuous or near continuous pain occurs in 14%–50% of patients.^1 13^ The importance of differentiating these two subtypes is underlined by recent evidence suggesting that TN with concomitant continuous pain is pathophysiologically different (see the Pathophysiology section) and responds less well to treatments compared with the purely paroxysmal form.

### Examination

The physical and neurological examinations are generally normal, though approximately 30% of cases can have sensory changes including mild hypoaesthesia.^7^ On rare occasions, during very severe attacks, the pain can evoke ipsilateral facial muscle contraction (tic douloureux).

## Pathophysiology

The current pathophysiological hypothesis for classical TN suggests that the pain mechanisms are precipitated by a proximal compression of the trigeminal sensory root near the brainstem (root entry zone) by a blood vessel (artery or vein). The root entry zone is considered a vulnerable area to demyelination, due to transition from the peripheral Schwann cell myelin sheath to central myelin generated by oligodendroglia. The vascular compression may start a process of focal demyelination and remyelination,^23 24^ probably mediated by microvascular ischaemic damages.^25^ These changes lower the excitability threshold of affected fibres and promote inappropriate ephaptic propagation towards adjacent fibres.^26^ Thus, tactile signals coming from the fast myelinated (A-β) fibres can directly activate the slow nociceptive (A-δ) fibres, resulting in the high-frequency paroxysms that characterise TN. After a few seconds, these repetitive discharges spontaneously run out and are followed by a brief period of inactivity that is called ‘refractory period’, where triggering actions cannot provoke pain.

The remarkable clinical effect of sodium channel blockers in TN has suggested that an abnormal expression of voltage-gated sodium channels could also constitute an important pathophysiological correlate for both classical and idiopathic TN, which might be sodium channelopathies. Nav1.7, Nav1.3 and Nav1.8 were found to be abnormally expressed in TN and possibly responsible for rapid activation and inactivation, as well as maintenance of the action potential.^27^ Over time hypersensitivity of tactile A-β fibres may lead to sensitisation of second-order wide dynamic range neurones in lamina V of the dorsal horns and the trigeminal nerve nuclei. Since these wide dynamic range neurones receive convergent information from tactile (A-β) and nociceptive (A-δ and C) fibres, their sensitisation could promote the perception of pain in response to cutaneous stimulation.

It was previously thought that TN with concomitant continuous pain occurred because of repetitive paroxysmal attacks. However, prospective cross-sectional studies show that the concomitant continuous pain often develops with or even before the onset of the paroxysmal pain.^13^ TN with concomitant persistent pain seems more prevalent in women and more often associated with sensory abnormalities than paroxysmal TN. Studies looking for impairment in trigeminal nociception have shown an abnormal nociceptive blink reflex and pain-related evoked potentials, indicating overactivation of central sensory transmission, as a potential mechanism to explain the constant facial pain of TN.^28^ Furthermore, an important recently published neuroimaging study using a 3T MR imaging of the trigeminal nerve roots in patients with ‘TN purely paroxysmal’ and ‘TN with concomitant continuous pain’ showed that the trigeminal nerve root was more severely atrophic in patients with concomitant continuous pain than in those with purely paroxysmal pain. The authors postulated that continuous pain most likely relates to axonal loss and abnormal activity in denervated trigeminal second-order neurones.^29^

## Differential diagnosis

TN is a clinical diagnosis based on detailed history and examination. Though often considered a straightforward diagnosis to make, its differential diagnosis can be challenging, given the considerable overlap with other neuropathic and neuralgiform headache and oro-facial pain disorders. Table 1 outlines the important differential diagnoses of TN.

**Table 1:** Differential diagnosis of trigeminal neuralgia

Dental causes	Dental caries Pulpitis Dental sensitivity Periodontal disorders Pericoronitis Cracked tooth Alveolar osteitis
Sinus causes	Maxillary sinusitis
Salivary gland causes	Salivary stone
Temporomandibular joint causes	Temporomandibular disorders
Neuropathic pain	Glossopharyngeal neuralgia Nervus intermedius neuralgia Post-herpetic neuralgia Post-traumatic trigeminal neuropathy Painful trigeminal neuropathies Atypical odontalgia Burning mouth syndrome
Trigeminal autonomic cephalalgias	SUNCT/SUNA Paroxysmal hemicrania Cluster headache Hemicrania continua
Other	Persistent idiopathic facial pain Primary stabbing headache

SUNA, short-lasting unilateral neuralgiform headache attacks with cranial autonomic symptoms; SUNCT, short-lasting unilateral neuralgiform headache attacks with conjunctival injection and tearing.

We have discussed below some selected differential diagnoses in greater detail as they often pose a challenge in neurological clinical practice.

### TN and the trigeminal autonomic cephalalgias

Since TN attacks are almost invariably precipitated by innocuous stimuli within the affected trigeminal distribution, all the trigeminal autonomic cephalalgias except SUNCT/SUNA can be easily differentiated from TN as none of the others can be triggered by innocuous stimuli. Recent studies on the demographics and clinical phenotype of SUNCT and SUNA have highlighted a remarkable overlap with TN.^20^ Furthermore, a recent prospective cross-sectional MR study conducted in 159 patients with SUNCT and SUNA showed a significantly higher proportion of neurovascular contact with morphological changes on the symptomatic trigeminal nerves, compared with the asymptomatic nerves. The multivariate analysis of radiological predictors associated with the symptomatic side indicated that the presence of neurovascular contact with morphological changes was strongly associated with the side of the pain, suggesting that this finding may be a shared causative factor with TN.^30^ A recent large prospective open-label study conducted in 161 patients on the medical treatments of SUNCT/SUNA confirmed the efficacy of sodium channel blockers, indicating also a therapeutic overlap with TN.^31^ Taken together these pieces of evidence suggest that SUNCT, SUNA and TN may constitute a continuum of the same disorder.^32^ Given the challenges in differentiating between them, table 2 summarises the differences between TN and SUNCT/SUNA.

**Table 2:** Clinical differences between trigeminal neuralgia and SUNCT/SUNA

Features	Trigeminal neuralgia	SUNCT/SUNA
Predominant pain distribution	V2/V3>V1	V1>V2/V3
Severity of pain	Very severe	Very severe
Duration (seconds)	<1–120	1–600
Autonomic features	None or sparse	Prominent
Spontaneous attacks only	None or rare	40%
Refractory period	Present	Absent
Periodicity	Mostly episodic	Mostly chronic
Preventive treatment of choice	Carbamazepine or oxcarbazepine	Lamotrigine

SUNA, short-lasting unilateral neuralgiform headache attacks with cranial autonomic symptoms; SUNCT, short-lasting unilateral neuralgiform headache attacks with conjunctival injection and tearing; V1, first division of the trigeminal nerve; V2, second division of the trigeminal nerve; V3, third division of the trigeminal nerve.

### TN and other forms of trigeminal neuropathic pain

The differential diagnosis between TN purely paroxysmal and other forms of trigeminal neuropathic pain is relatively straightforward in view of the lack of the constant facial pain component in TN purely paroxysmal, whereas other trigeminal neuropathic pain disorders are characterised by a constant dull, aching, burning and/or throbbing pain. However, it is more challenging to distinguish between TN with concomitant persistent pain and trigeminal neuropathic pain conditions. Table 3 outlines some useful clinical differences between these conditions.

**Table 3:** Differential diagnosis between trigeminal neuralgia (TN) with concomitant facial pain and other trigeminal neuropathic conditions

Features	TN with concomitant persistent facial pain	Idiopathic neuropathic pain*	Neuropathic pain with identifiable cause†	Persistent idiopathic facial pain
Precipitating factor	No	No	Yes (trauma, viral, inflammatory)	No (possible stress)
Pain location	Extra/intraoral	Extra/intraoral	Extra/intraoral	Extraoral
Laterality and trigeminal distribution	Unilateral Dermatomal	Unilateral Dermatomal	Unilateral Dermatomal	Often bilateral Non-dermatomal
Pain severity	Severe–very severe	Mild to severe	Mild to severe	Mild to severe
Other sensory symptoms	None	Yes	Yes	None
Cutaneous/intraoral triggers	Present	Yes, but rare	Present	None
Effective treatments	Carbamazepine	Tricyclic antidepressants, gabapentinoids	Tricyclic antidepressants, gabapentinoids	Unclear

*Includes persistent dentoalveolar pain, atypical odontalgia, phantom tooth pain in which the pain location is intraoral only.

†This term mainly includes painful post-traumatic trigeminal neuropathy and post-herpetic neuropathic pain.

When phenotyping a patient with trigeminal neuropathic pain, it is important to highlight that a pivotal feature of TN is stimulus-evoked pain by innocuous mechanical stimuli within the trigeminal territory, including the oral cavity. The cutaneous triggerability of the attacks in TN differs from allodynia in that the trigger zones and pain sensation may be dissociated in TN but not in allodynia. This phenomenon has been suggested to represent a sign of cross-excitation between somatosensory afferents.^33^

## Investigations

MR of the brain is the gold-standard investigation to exclude secondary causes of TN. If MR is contraindicated, a CT scan of the head, CT cerebral angiogram and trigeminal-evoked potentials and/or neurophysiological recordings of trigeminal reflexes should be used.^9^

Besides excluding secondary TN, neuroimaging is also important for further subclassifying a patient’s symptoms into classical and idiopathic TN, so that the classical TN cases can be considered for trigeminal microvascular decompression when appropriate. Detailed trigeminal MR brain scan sequences are pivotal to detecting the presence of a trigeminal neurovascular conflict, the type of vascular structure (artery or vein or both) and the degree of compression. The protocol should use the combination of three high-resolution sequences that include a 3D cisternal fast imaging employing steady-state acquisition, constructive interference in steady state or sampling perfection with application optimised contrasts using different flip angle evolution sequences along with time-of-flight MR-angiography as well as 3D T1-weighted gadolinium sequences.^34^

## Treatment

Abortive treatments are not useful in the management of TN due to the brevity of the attacks. The mainstay of management is pharmacological preventive treatments. However, acute treatments that work rapidly have to be used occasionally for severe exacerbation. Surgical interventions are reserved for patients who fail to respond to or adequately tolerate medical therapies.

### Pharmacological preventive treatments

The arsenal of preventive treatments for TN has now been in use for several decades but the quality of the evidence base is poor and there are few high-quality randomised controlled trials. Though these treatments are not supported by good quality randomised controlled trials, the clinical experience with some of these drugs (particularly carbamazepine, oxcarbazepine, lamotrigine, gabapentin, pregabalin, baclofen and botulinum toxin type A) is good, resulting in meaningful pain control although with still a substantial unmet need for more effective and better tolerated drugs. Table 4 summarises the available data on the commonly used drugs. Table 5 outlines the preventive treatments, recommended doses, and titration and tapering regimens we use. While monotherapy is preferred, up to one-third of patients require polytherapy, emphasising these patients’ unmet therapeutic need.^35^ Patients should be encouraged to keep pain diaries to enable monitoring of response to treatments.

**Table 4:** Summary of randomised controlled trials for pharmacological treatments in trigeminal neuralgia

	Number of RCTs	Number of patients	Dose range (mg/day)	Responder rate
Carbamazepine	3	138	800–1200	68%–100%
Oxcarbazepine	1	48	600–1800	100%
Lamotrigine	1	14	200–400	85%
Gabapentin*	16	1156	Up to 3600	Reportedly similar to carbamazepine
Baclofen	1	10	30–60	70%
Botulinum toxin type A	4	178	25–100 units	68%–86%
Pimozide	1	48	4–12	100%†
Tizanidine	1	12	18	20%

Pregabalin: no RCTs available.

*Gabapentin: all RCTs are in Chinese language and results difficult to access.

†Pimozide trial has been heavily criticised for methodological pitfalls.

RCTs, randomised controlled trials.

**Table 5:** Preventive treatments in trigeminal neuralgia (adapted from Bendtsen *et al* 41)

Drug	Initiating dose	Titration*	Dose range	Frequency	Tapering†	Specific side effects
Carbamazepine	200 mg	200 mg every 3 days	200–1200 mg	Two to four times per day	200 mg every 7 days	Dizziness, drowsiness, fatigue, ataxia, diplopia, nausea, cognitive slowing, hyponatraemia leucopenia, thrombocytopenia, skin reactions, abnormal liver function tests
Oxcarbazepine	300 mg	300 mg every 3 days	300–1800 mg	Four times per day	300 mg every 7 days	Dizziness, drowsiness, fatigue, nausea, ataxia, hyponatraemia, skin reaction
Lamotrigine	25 mg	25 mg for 2 weeks, 50 mg for 1 week, then increase by 50 mg every week	25–400 mg	Two times per day	50 mg every 7 days	Dizziness, drowsiness, fatigue, headache, gastrointestinal symptoms, irritability, sleep disorders, tremor, cognitive slowing, rash
Gabapentin	300 mg	300 mg every 3 days	300–3600 mg	Three times per day	300 mg every 7 days	Dizziness, confusion, fatigue, ataxia, increased risk of infection, gastrointestinal symptoms, weight gain; use cautiously with opioids
Pregabalin	150 mg	150 mg every 7 days	150–600 mg	Two times per day	100 mg every 7 days	Dizziness, confusion, ataxia, increased risk of infection, gastrointestinal symptoms, weight gain
Baclofen	15 mg	15 mg every 7 days	15–90 mg	Three times per day	15 mg every 7 days	Confusion, dizziness, drowsiness, gastrointestinal symptoms, euphoria, hallucinations
Botulinum toxin type A	25–195 units	NA	25–195 units	Every 12 weeks	NA	Transient facial asymmetry, transient bruising at injection site, transient drooling and difficulty chewing

*The doses can be increased at a slower rate to improve tolerability; the doses are increased until the pain is well controlled, significant side effects intervene or the maximum dose is achieved.

†The doses can be reduced more gradually and in smaller decrements; the dose is reduced to either the minimum required to control the pain or allow cessation of drug without recurrence of pain.

NA, not applicable.

#### Carbamazepine and oxcarbazepine

Carbamazepine and oxcarbazepine are the first-line treatment options for TN and offer meaningful initial pain control in almost 90% of patients,^19^ although this may not be sustained in the long term. The benefit of these drugs is offset by adverse effects, which lead to withdrawal in up to 40% of patients.^35^ Carbamazepine is known for its metabolic interaction with other medications, which can be problematic in elderly people with comorbidities. Oxcarbazepine causes fewer side effects and has lower potential for drug interactions than carbamazepine, though it is more likely to cause excessive central nervous system depression or dose-related hyponatraemia. The tolerability of both these drugs is gender related; women are significantly less tolerant.

The individual response to both drugs varies considerably, hence if one is not effective, then the other one can be tried. If changing over from carbamazepine to oxcarbazepine, then 200 mg of carbamazepine is equipotent to 300 mg of oxcarbazepine. It is important to be aware that the modified-release (retard) version of carbamazepine available is best used when patients have stabilised. Liquid versions of both drugs are useful when patients find it hard to swallow due to pain severity. While these drugs are effective for control of the paroxysmal pain, their effect on the concomitant continuous pain is usually limited.

Contraindications to using these agents include cardiac conduction problems and allergic reactions. There is a high degree of cross-reactivity between the aromatic antiseizure medications (carbamazepine, oxcarbazepine, phenytoin, phenobarbital).

Carbamazepine and oxcarbazepine do not generally require regular monitoring of serum drug concentrations; in most patients, the drug doses can be titrated or tapered by clinically considering the balance between the efficacy and adverse effects. However, we advocate regular monitoring of renal, calcium and liver function tests. Patients may develop hyponatraemia and a cholestatic picture on liver function testing which, while not usually of clinical concern, need careful monitoring to ensure that they do not progressively worsen. Older women are already at increased risk of osteoporosis and this needs to be monitored in long-term use. The HLA-B*1502 allele is highly associated with the outcome of carbamazepine-induced Stevens-Johnson syndrome and toxic epidermal necrolysis. This association has been found mostly in the Han Chinese, but not in Caucasian patients. Hence, all Han Chinese patients should be tested for this allele before starting carbamazepine.

#### Lamotrigine

Lamotrigine has been reported to be helpful as an add-on therapy in a small randomised cross-over trial.^36^ Lamotrigine can be used in patients who cannot tolerate carbamazepine and oxcarbazepine, or as add-on therapy to increase efficacy. It is generally associated with fewer side effects than carbamazepine and oxcarbazepine. The dose of lamotrigine should be escalated slowly as the incidence of lamotrigine-induced rash is well recognised to be dose and titration dependent. About 10% of people taking lamotrigine develop benign adverse cutaneous reactions. However life-threatening conditions, like Stevens-Johnson syndrome, can rarely occur. Since the introduction of a slow-dose titration protocol, the rate of severe rashes has reduced to 0.1%–0.01%.^37^ In view of the need for this slow-dose titration, lamotrigine is not appropriate for managing severe TN exacerbation to those who need rapid pain control.

#### Gabapentin and pregabalin

There are 16 randomised controlled trials for gabapentin, all published in Chinese, comparing it with carbamazepine. However, it is difficult to draw any meaningful conclusions as the inclusion criteria, endpoints and dosage are either not clarified or very varied. There are no such trials for pregabalin, but a long-term study suggests that it may be effective.^38^

Clinical experience shows that gabapentin and pregabalin are less effective but have fewer side effects than carbamazepine and oxcarbazepine. They can therefore be used in place of or in addition to carbamazepine or oxcarbazepine. However, there is a risk of dependency and they are controlled drugs in the UK.

#### Baclofen

Baclofen can help in TN especially in people with multiple sclerosis who may be using the drug for spasticity.

#### Botulinum toxin type A

Recent randomised controlled trials of botulinum toxin type A have provided evidence for efficacy in TN. The botulinum toxin type A was injected subcutaneously and occasionally over the gingival mucosa. The dose varies among trials between 25 and 100 units applied following the pain distribution, 1 cm apart, often for a total of 10–20 injection points. Most trial outcomes were evaluated at 3 months. All trials showed consistent significant superiority of botulinum toxin type A compared with placebo. Responders to botulinum toxin type A ranged between 68% and 86% compared with 15%–32% of placebo. Adverse effects were mild to moderate and included transient facial weakness and transient facial oedema. Overall, these studies point towards a clear efficacy of botulinum toxin type A in TN.

#### Other treatments

Other drugs reported in small open-labelled studies include phenytoin, tizanidine, levetiracetam, misoprostol (especially in patients with multiple sclerosis), topiramate, pimozide, duloxetine and eslicarbazepine. A novel sodium channel blocker, vixotrigine, has been tested in one randomised controlled trial and phase three trials are due to start shortly.

### Acute treatment for severe exacerbation

Severe exacerbation during which there is a marked increase in the frequency and intensity of pain, resulting in an inability to eat or drink and may require admission to hospital for rehydration, maintenance of nutrition, short-term pain management and long-term optimisation of preventive treatments. Though opioids are frequently used, they are generally ineffective and should be avoided. Topical lidocaine or local anaesthetic injections into the trigger zones can provide transient relief.^39^ Intravenous infusions of fosphenytoin (15 mg/kg over 30 min) and lidocaine (5 mg/kg over 60 min) under cardiac monitoring can be highly effective but should be administered by specialised teams with expertise in their use and in the setting of a high dependency unit.^39^

### Surgical treatments

Surgical treatments are generally reserved for patients with debilitating pain refractory to pharmacological treatments. There are three types of surgical intervention available: (1) invasive, non-ablative (microvascular decompression), (2) invasive, ablative (controlled lesioning of the trigeminal ganglion or root by mechanical (balloon compression), thermal (radiofrequency thermocoagulation) or chemical means (glycerol rhizolysis), separation of trigeminal nerve fascicles in the posterior fossa (internal neurolysis)) and (3) non-invasive ablative (stereotactic radiosurgery which focuses radiation at the trigeminal root entry zone). Table 6 outlines the efficacy and complications of the various procedures. The diagnostic criteria and outcome measures used in the neurosurgery reports are very varied and make comparisons exceedingly difficult.

**Table 6:** Surgical intervention for trigeminal neuralgia^34^

Intervention	Microvascular decompression	Stereotactic radiosurgery	Radiofrequency thermocoagulation	Balloon compression	Glycerol rhizolysis	Internal neurolysis
Efficacy data						
Number of studies	21	8	7	5	3	1
Total number of patients	5149	1168	4533	755	289	26
Mean/median follow-up	3–10.9 years	3.1–5.6 years	3–9.3 years	4.2–10.7 years	4.5–8 years	3.6 years
Pain free at follow-up (5)	62%–89%	30%–66%	26%–82%	55%–80%	19%–58%	72%
Complications (%)						
Facial sensory changes	3	16	19	15	40	96
Corneal hypoaesthesia	0.3	0	6.6	0.7	6.6	0
Hearing loss	1.8	0	0.1	0	0.3	0
Motor weakness	0	0	6.2	4.5	1.7	0
Cranial nerve palsy	4.1	0.2	0.8	1.6	0	0
Meningitis	0.4	0	0.02	5.7	0	0
CSF leak	2	0	0.1	0	0	3.8
Anaesthesia dolorosa	0.02	0	0.6	0.1	0.7	3.9
Mortality	0.3	0	0	0	0	0

CSF, cerebrospinal fluid.

**Figure 2:**
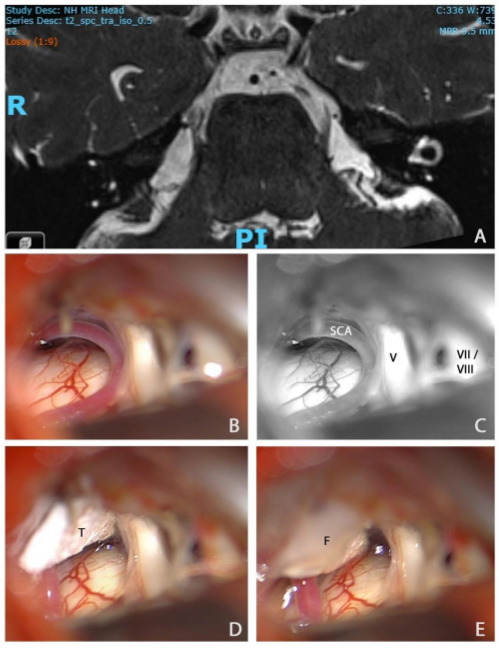
MR scan of the trigeminal nerve and intraoperative pictures during microvascular decompression in patient with classical trigeminal neuralgia. (A) Axial MR 0.5 mm volumetric SPACE sequence through the pons showing neurovascular conflict between the right superior cerebellar artery (SCA) and the right trigeminal nerve (V). (B) Intraoperative view of the right cerebellopontine angle, prior to right microvascular decompression, showing conflict between the right SCA and V. (C) Black and white rendition of the previous photograph with labelling of the superior cerebellar artery, V, and more superficial seventh and eighth nerve complex (VII/VIII). (D) The superior cerebellar artery has been mobilised and transposed superiorly towards the tentorium. It is held in place with a small piece of Teflon (T). (E) A small drop of fibrin glue (F) has been applied to ensure that the T does not migrate. A small ‘dent’ in the course of the trigeminal nerve can be seen at the site of the previous neurovascular conflict. SPACE, sampling perfection with application optimised contrasts using different flip angle evolution.

Microvascular decompression is the surgery of first choice in classical TN (see figure 2). Data in over 5000 patients showed a pain-free rate of 62%–89% after 3–10 years of follow-up.^34^ The annual risk of recurrence is less than 2% 5 years after the operation and less than 1% after 10 years. TN with concomitant continuous pain has poorer outcome, with pain freedom rates dropping to 23.5%–51% at 5 years of follow-up, although that is not a consistent finding. While previous studies of microvascular decompression did not distinguish effectively between classical and idiopathic TN, the emerging evidence unsurprisingly suggests that it is more effective in classical than idiopathic TN. The data on decompression in TN secondary to multiple sclerosis are conflicting. The responder rates in the published series varied between 39% and 100% with follow-up periods of 12–65 months. The general advice in these patients would be to consider microvascular decompression if the MR scan shows morphological changes and in absence of a plaque in the pons, given that very recent evidence suggested that a brainstem lesion related to the TN on MR is a negative prognostic factor for microvascular decompression.^40^ Trigeminal microvascular decompression is a major procedure that can be carried out successfully in the elderly provided they have no significant comorbidities, but results are poorer in those younger than 25 years. Severe complications are rare but there is small risk of mortality (0.3%).

When there is no evidence of trigeminal neurovascular contact or there are significant comorbidities, ablative procedures are the preferred choice. The least invasive procedure is stereotactic radiosurgery. However, pain relief can be delayed by up to 6 months and sensory loss occurs frequently. Emerging evidence suggests that trigeminal internal neurolysis is highly effective in the long term but has a high complication rate (facial hypoaesthesia 96%, anaesthesia dolorosa 3.9%). The percutaneous neuroablative procedures (radiofrequency thermocoagulation, balloon compression, glycerol rhizolysis) provide on average 3–4 years of pain relief and repetitive ablative procedures are commonly required. Complication rates are high, especially with repetitive procedures. There is no evidence for preference of one procedure over another.

**Figure 3:**
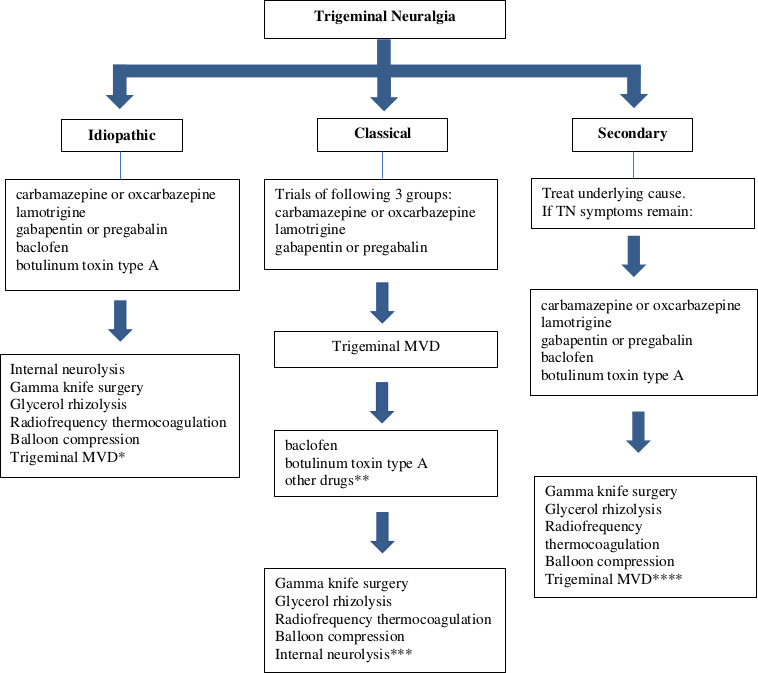
Proposed algorithm for the treatment of trigeminal neuralgia. *Microvascular decompression (MVD) appears effective even in idiopathic TN and may be more effective than stereotactic radiosurgery. **If any of carbamazepine, oxcarbazepine, lamotrigine, gabapentin or pregabalin have not been tried then trials of these agents should be considered. ***Internal neurolysis is best avoided after MVD as there is a suggestion that there is a higher risk of anaesthesia dolorosa. ****Consider MVD if the secondary cause is optimally treated and there is evidence of neurovascular conflict; exercise caution in MS with ipsilateral pontine plaque. MS, multiple sclerosis; TN, trigeminal neuralgia.

There is no clear guidance on the number of medical treatments that a patient has to fail before surgical approaches should be offered. It is important to make patients aware of the management options available, including both the medical and surgical approaches, early in the treatment pathway. We have outlined an algorithm of our practice (see figure 3). In patients with classical TN, we consider microvascular decompression when patients report a poor quality of life and there is either failure to respond or significant adverse effects with up to three groups of drugs. Carbamazepine and/or oxcarbazepine followed by lamotrigine and a gabapentinoid (gabapentin or pregabalin) can be tried. These can be used in combination. If these patients fail to respond to microvascular decompression, then we offer trials of other drugs not tried until then before considering neuroablative procedures. In both idiopathic and secondary TN (without evidence of neurovascular conflict), we tend to try more pharmacological treatments before considering neuroablative procedures, mainly because of the risk of long-term complications particularly with repetitive percutaneous neuroablative procedures. Patients who develop superimposed severe trigeminal neuropathy secondary to the neuroablative procedures can be very challenging to manage in the long term.

### Other considerations

Patients with TN, especially those whose symptoms are proving refractory to pharmacotherapy, are best managed in multidisciplinary team setting with a neurologist specialising in headache disorders, pain specialist, neurosurgeon, nurses and psychologists.^35^ In particular, we suggest that neurosurgical procedures for TN should only be done by experts with a high volume of cases to maintain the neurosurgical experience. Patients may benefit from a pain management programme to help them live well with pain and uncertainty.

Due to the rarity of the condition, patients feel isolated and patient groups such as Trigeminal Neuralgia Association TNA UK provide further invaluable support. Patients need to be provided with evidence-based written information such as provided by the Brain and Spine Foundation and advice from skilled teams.

## Prognosis

TN is characterised by recurrences and remissions. Many people have periods of remission with no pain lasting months or years but in many, TN becomes more severe and less responsive to treatment over time, despite increasing pharmacological intervention. Most patients with TN are initially managed medically, and at our tertiary referral centre approximately 50% eventually have a surgical procedure.^35^

## Conclusions

Recent advances in TN have led to an improvement in its classification on the basis of the neuroimaging findings. Better understanding and description of other neuralgiform disorders such as SUNCT and SUNA have made the differential diagnosis clearer. Improved care pathways involving multidisciplinary teams and potentially new medications is resulting in improved outcomes for patients with TN.

**Key points**

- Trigeminal neuralgia is currently classified into three subgroups: idiopathic, classical and secondary, based on imaging findings; MR brain imaging with trigeminal sequences is therefore essential in the diagnostic work-up.
- An accurate diagnosis is crucial because the clinical management differs among the various forms of facial pain.
- Carbamazepine and oxcarbazepine remain the medications of choice; lamotrigine, gabapentin, pregabalin, botulinum toxin type A and baclofen can be used as second-line treatments in monotherapy or polytherapy.
- In pharmaco-resistant cases, trigeminal microvascular decompression is the first-line surgery in patients with classical trigeminal neuralgia, whereas neuroablative surgical treatments and microvascular decompression can be considered in idiopathic trigeminal neuralgia.
- Pharmaco-resistant cases as well as cases where diagnosis is unclear should be referred to multidisciplinary facial pain teams led by neurologists specialising in headache disorders, where dedicated teams may confirm the diagnosis and offer advanced treatments.

**Further reading**

1. Cruccu G, Di Stefano G, Truini A. Trigeminal Neuralgia. *N Engl J Med*. 2020;383 :754–62.
2. Bendtsen L, Zakrzewska JM, Heinskou TB, Hodaie M, Leal PRL, Nurmikko T, *et al*. Advances in diagnosis, classification, pathophysiology, and management of trigeminal neuralgia. *Lancet Neurol*. 2020;19 :784–96.
